# *“Why Would Someone like Me with DLD Want to Sit in a Room and Talk? How Would that Make Me Feel Better?!”* Developmental Language Disorder and the Language Demands of Cognitive Behaviour Therapy

**DOI:** 10.1007/s41811-025-00254-3

**Published:** 2025-05-27

**Authors:** Elizabeth Hill, Kate Tonta, Mark Boyes, Courtenay Norbury, Sarah Griffiths, Shaun Goh, Brooke Ryan

**Affiliations:** 1 Curtin School of Allied Health, Curtin University, Perth, Australia; 2 Curtin enAble Institute, Curtin University, Northbridge, Australia; 3 Centre for Clinical Interventions, Northbridge, Australia; 4 Curtin School of Population Health, Curtin University, Perth, Australia; 5 University College London Division of Psychology and Language Sciences, London, UK; 6 Department of Special Needs Education, University of Oslo, Oslo, Norway; 7 Nanyang Technological University National Institute of Education, Singapore, Singapore

**Keywords:** Language difficulties, Accessibility, Cognitive behaviour therapy, Developmental language disorder

## Abstract

This article addresses two crucial questions: what are the language demands of cognitive behavioural therapy (CBT), and how can CBT be more accessible to people with language difficulties? These questions are examined through a case study of Developmental Language Disorder (DLD). With approximately 7% of children living with DLD, mental health practitioners have inevitably encountered someone with this condition. Many children with behavioural and emotional problems have unidentified language difficulties, increasing their risk of mental health problems. Language is the medium through which CBT is delivered, so exploring its language-based accessibility is of critical concern. This paper (i) describes a language framework, (ii) identifies the language skills required for CBT engagement, and (iii) outlines strategies to improve language-based accessibility. These considerations are conceptualised in the context of DLD, yet they apply to any neurodivergent population with language needs who may benefit from CBT.

**Supplementary Information:**

The online version contains supplementary material available at 10.1007/s41811-025-00254-3.

## Introduction

The goal of this paper is to reveal the language demands of Cognitive Behaviour Therapy (CBT). To scaffold our discussion, we have framed this theoretical paper around a case study of developmental language disorder (DLD). DLD is the most common neurodevelopmental condition, affecting 1 in 14 children, and places a child at substantially elevated risk of mental health difficulties (Norbury et al., 2016; Yew & O’Kearney, 2013). We introduce Taylor, a child with unidentified DLD and depressive symptoms, whose psychological therapy was discontinued due to disengagement. We argue that one reason for Taylor’s disengagement may be a misalignment between her language skills and the language demands of CBT (Hobson et al., 2022). This misalignment highlights the need for mental health professionals to be aware of the language demands of CBT and to adapt their approaches accordingly. Ideally, in cases such as Taylor, mental health professionals should recognise language difficulties and refer to language specialists (e.g., speech-language therapists) promptly. However, regardless of whether specialist language intervention is in place, individuals with co-occurring language and mental health challenges require accessible mental health support. In the context of Taylor’s case, we do not suggest that her mental health practitioner should have treated her language difficulties in the absence of a formal diagnosis of language disorder or language specialist involvement. Rather, we aim to highlight the language demands of CBT and guide mental health professionals to adapt the content and delivery of therapy to ensure language-based accessibility.

To illustrate this argument, we describe a framework for language analysis and proceed to identify and outline the language skills required to engage in CBT. The paper concludes with a description of strategies and potential adaptations that may support participation. We emphasise that, while DLD is the context of this theoretical paper, the language demands are likely to impact engagement for a range of populations with language difficulties, for example, autistic individuals with language needs. Mental health professionals must understand these language demands as a starting point, so they can make mental health therapies, such as CBT, accessible for individuals with co-occurring language difficulties.

## Case Study: Taylor

Taylor is a 14-year-old girl who was referred to child and adolescent mental health services (CAMHS). Since turning 13, Taylor’s teachers have raised concerns about her behaviour and her academic performance, which have noticeably deteriorated in the last 12 months. Taylor has been described as distracted and disinterested in class and her family believes that she has not enjoyed what she has been learning. More recently, Taylor stopped socialising with her friends, she has been sleeping in every morning and refusing to go to school. Taylor’s referral to CAMHS was her first time receiving psychological care, and Taylor’s psychologist implemented CBT for depressive symptoms as a first line intervention. The psychologist found it difficult to connect with Taylor; it seemed as if Taylor was not paying attention during sessions, and she did not provide many details about how she was feeling and why. Taylor attended sessions for three weeks before becoming disengaged and refusing to attend. Consequently, her parents made the decision to discontinue psychological support.

Underpinning Taylor’s social and academic experiences are underlying difficulties with her use and understanding of language. While she is fully intelligible and pronounces sounds and words correctly, Taylor finds it hard to comprehend others and express herself clearly to her family and friends because of limited vocabulary and grammar. She speaks more slowly than her peers, and it takes longer for her to respond to questions, particularly in a distracting environment like her classroom. During conversations with friends and family, Taylor often leaves out key contextual details, such as who is involved or exactly what happened. Completing everyday tasks at home and at school can also be difficult for Taylor because she has trouble following multiple-step complex directions. Taylor’s language difficulties are particularly noticeable when she is tired, speaking with unfamiliar people or about unfamiliar topics, and when she is stressed or anxious.

Taylor’s language difficulties are consistent with developmental language disorder (DLD). However, like many girls who experience persistent and pervasive language difficulties, Taylor has never received a formal diagnosis, so her mental health practitioner is likely to be unaware of her language difficulties. Indeed, many of Taylor’s social and academic concerns are common among children with language problems as skilled use and understanding of language are necessary to participate wholly in every aspect of daily life (St Clair et al., 2019; Ziegenfusz et al., 2024). Language facilitates social and emotional development, the development and maintenance of relationships, and the acquisition and demonstration of knowledge. It is therefore unsurprising that there is growing evidence that language difficulties significantly increase the risk of poor mental health outcomes (St Clair et al., 2011; Yew & O’Kearney, 2013). Given the over-representation of children with language difficulties in referrals to child and adolescent psychiatry services (Cohen et al., 2013) it is noteworthy that Taylor’s language challenges have adversely affected her capacity to engage in the complex, verbal interactions required in CBT. In addition, given that she does not know how to describe her challenges with talking and listening, it is unlikely that Taylor has learnt ways to advocate for her language needs and inform her therapist that she is having trouble understanding what is being asked of her.

## What is Developmental Language Disorder?

There have been many labels to describe child language disorder that is not attributed to another biomedical condition (e.g., autism or intellectual disability), including ‘language disorders of unknown origin’ (Norbury et al., 2016) and ‘specific language impairment’ (Tomblin et al., 1997). This lack of consensus regarding terminology for significant and persistent early language difficulties triggered a multidisciplinary and international consensus process (Bishop et al., 2017). This exercise proposed DLD as the standard label that aligns with contemporary international classifications for disability (Table 1 provides an overview of contemporary diagnostic criteria).

**Table 1:** Contemporary diagnostic criteria for (Developmental) Language Disorder

Criterion	CATALISE^1^	ICD-11^2^	DSM-5, 315:39(F80.9)^3^
Persistence	Research has shown children are unlikely to catch up to peers spontaneously	Persistent deficits in the acquisition, understanding, production or use of language (spoken or signed) The individual’s ability to understand, produce or use language is markedly below what would be expected given the individual’s age	Persistent difficulties in the acquisition and use of language across modalities (i.e., spoken, written, or other) due to deficits in comprehension or production that include the following: • Reduced vocabulary (word knowledge and use) • Limited sentence structure (ability to put words and word endings together to form sentences based on the rules of grammar and morphology) Impairments in discourse (ability to use vocabulary and connect sentences to explain or describe a topic or series of events or have a conversation)
Function	Language difficulties that create obstacles to communication or learning in everyday life	Cause significant limitations in the individual’s ability to communicate	Language abilities are substantially and quantifiably below those expected for age, resulting in functional limitations in effective communication, social participation, academic achievement, or occupational performance, individually or in any combination
Early onset	Onset of symptoms is in early childhood, and neurobiological (e.g., male) and environmental (e.g., poverty) risk factors do not preclude a diagnosis	Onset of symptoms is in the early developmental period	Onset of symptoms is in the early developmental period
Exclusion	Language disorder is not associated with a known biomedical condition (such as: brain injury, acquired epileptic aphasia in childhood, certain neurodegenerative conditions, genetic conditions such as Down Syndrome, cerebral palsy, sensorineural hearing loss, autism spectrum disorder, intellectual disability)	The language deficits are not explained by another neurodevelopmental disorder or a sensory impairment or neurological condition, including the effects of brain injury or infection (exclusions: autism spectrum disorder (6 A02), disease of the nervous system (8 A00-8E7Z), Deafness not otherwise specified (AB52), selective mutism (6B06))	The difficulties are not attributable to hearing or other sensory impairment, motor dysfunction, or another medical or neurological condition and are not better explained by intellectual disability (intellectual developmental disorder) or global developmental delay

Table adapted from Calder et al. (2023). ^1^Bishop et al. (2017). ^2^ World Health Organisation (2019). ^3^American Psychiatric Association (2013)

DLD is ‘the most common neuro-developmental disorder you’ve never heard of’ (Norbury, 2017). Seven times more common than autism, estimates of the prevalence of DLD in Australia (Calder et al., 2022), China (Wu et al., 2023), the UK (Norbury et al., 2016), and the USA (Tomblin et al., 1997) indicate approximately 7% of children meet diagnostic criteria (57–59% male). While some characteristics of DLD are language specific, universal indicators of the disorder include slower acquisition of language, grammatical errors, poor auditory working memory, difficulty following instructions, and poor topic maintenance, as well as less sophisticated or non-specific vocabulary, word-finding difficulties, and shorter or less mature sentences (APA, 2013; Bishop et al., 2017). These language difficulties are persistent and pervasive; they can impact participation at school, social outcomes, and wellbeing (Conti-Ramsden et al., 2019; Goh et al., 2021; Ziegenfusz et al., 2024). Critically, DLD is a *lifelong* neurodevelopmental condition, contributing to poorer vocational and socioeconomic outcomes in late adolescence and adulthood (Botting, 2020; Conti-Ramsden & Durkin, 2012; McGregor, 2020; Nippold & Schwarz, 2002; Whitehouse et al., 2009).

The language difficulties associated with DLD are biologically based; that is, they are not attributed to lack of exposure to language or inadequate educational instruction (Bishop et al., 2017). DLD is typically diagnosed and managed by qualified speech-language therapists (SLTs), who are responsible for and qualified to support the primary difficulties (i.e., language and communication) associated with the condition. SLTs can work with people with DLD to target a range of language and communication skills, including grammar and vocabulary, complex social communication skills (e.g., conversation, story-telling, making inferences), and the language required for academic and vocational participation. Though DLD cannot be ‘cured’, SLT intervention is effective in improving these discrete aspects of language competence (Donolato et al., 2023; Fan et al., 2022; Law et al., 2004; Roberts & Kaiser, 2011). However, intervention provided by SLTs is unlikely to resolve co-occurring difficulties, particularly those related to socioemotional and behavioural wellbeing (Hobson et al., 2022).

Returning to Taylor’s case, in the absence of specialist language intervention, one may assume that she may benefit from explicit, evidence-informed language support embedded within good universal educational practices. In some instances, modified educational practices may inadvertently widen the academic gap between children with and without language disorder by failing to adequately address their specific needs (Duff et al., 2023; West et al., 2024; Ziegenfusz et al., 2024). Therefore, regardless of whether a child with DLD receives specialist intervention or adapted educational support, modifications to mental health interventions will be necessary to ensure they are accessible.

Compared to their neurotypical peers, people with DLD are approximately twice as likely to show clinical levels of emotional and behavioural problems. This population tends to be above the 70 th percentile in severity of emotional problems, with DLD associated with substantially elevated risk of anxiety disorders, depression, schizotypal features, conduct problems, and incarceration throughout child- and into adulthood (Botting et al., 2016; Clegg et al., 2005; Conti-Ramsden & Botting, 2008; St Clair et al., 2011; van den Bedem et al., 2018; Winstanley et al., 2018; Yew & O’Kearney, 2013). Children who have another developmental condition in addition to DLD are at even greater risk of mental health challenges (e.g., Hannig Russel & Remond, 2024). It is also critical to note that as many as 80% of young people with emotional and/or behavioural disorders have unidentified language difficulties (Hollo et al., 2014), with common mental health diagnoses (e.g., anxiety disorder) associated with *subclinical* language difficulties (Sbicigo et al., 2020). Combined, children with co-occurring language and mental health challenges are statistically likely to make up a considerable proportion of those attempting to access and engage in mental health services.

Language plays a significant role in mental health. Our vocabulary allows us to label and express thoughts and emotions. How we order words (syntax) allows us to articulate the relationships between people, places, events, and/or emotions, such as the causal or temporal links between them. Sentences can be arranged in different ways to tell stories, express opinions, and argue perspectives, all of which facilitate social interaction, support emotional regulation (e.g., self-talk) and inform understanding of our own and others’ experiences. Language is a powerful tool that underpins the way in which we make sense of, reflect on, and communicate our experience of the world around us. Consequently, it is not surprising that language difficulties are associated with increased risk of mental health difficulties and trouble accessing mental health support.

Despite this link, the degree to which language intervention benefits mental health outcomes is unknown. There is evidence of temporal precedence, where early language development predicts later emotion processing and regulation (Bell et al., 2024; Forrest et al., 2020; Griffiths, et al., 2021). These constructs are, in turn, associated with parent ratings of child anxiety and depression (Schneider et al., 2016; Young et al., 2019). This observation sets up the hypothesis that improvements in language skills could have cascading impacts on mental health (and vice versa), although this requires ongoing investigation (Curtis et al., 2019; West et al., 2024). Other mechanisms may include increased self-esteem, advocacy, and improvements in language facilitating the development and maintenance of social networks. Until such studies are undertaken, children with DLD and co-occurring mental health difficulties will require two separate and ideally coordinated, treatment pathways — language intervention and mental health intervention (Hobson et al., 2022).

Currently, most mental health interventions are language-intensive and mediated by verbal interactions, reading, and writing. Consequently, psychotherapies or, ‘talking therapies’ for mental health difficulties are generally not accessible to children with language difficulties (Hobson et al., 2022; Norbury et al., 2024). The provision of evidence-based mental health interventions that accommodate for language difficulties has been recognised as an urgent priority for research and clinical practice (Hobson et al., 2022). An important, and necessary, step towards this is to explicitly consider the language demands associated with evidence-based psychotherapies such as CBT.

## Cognitive Behaviour Therapy

**Fig. 1:**
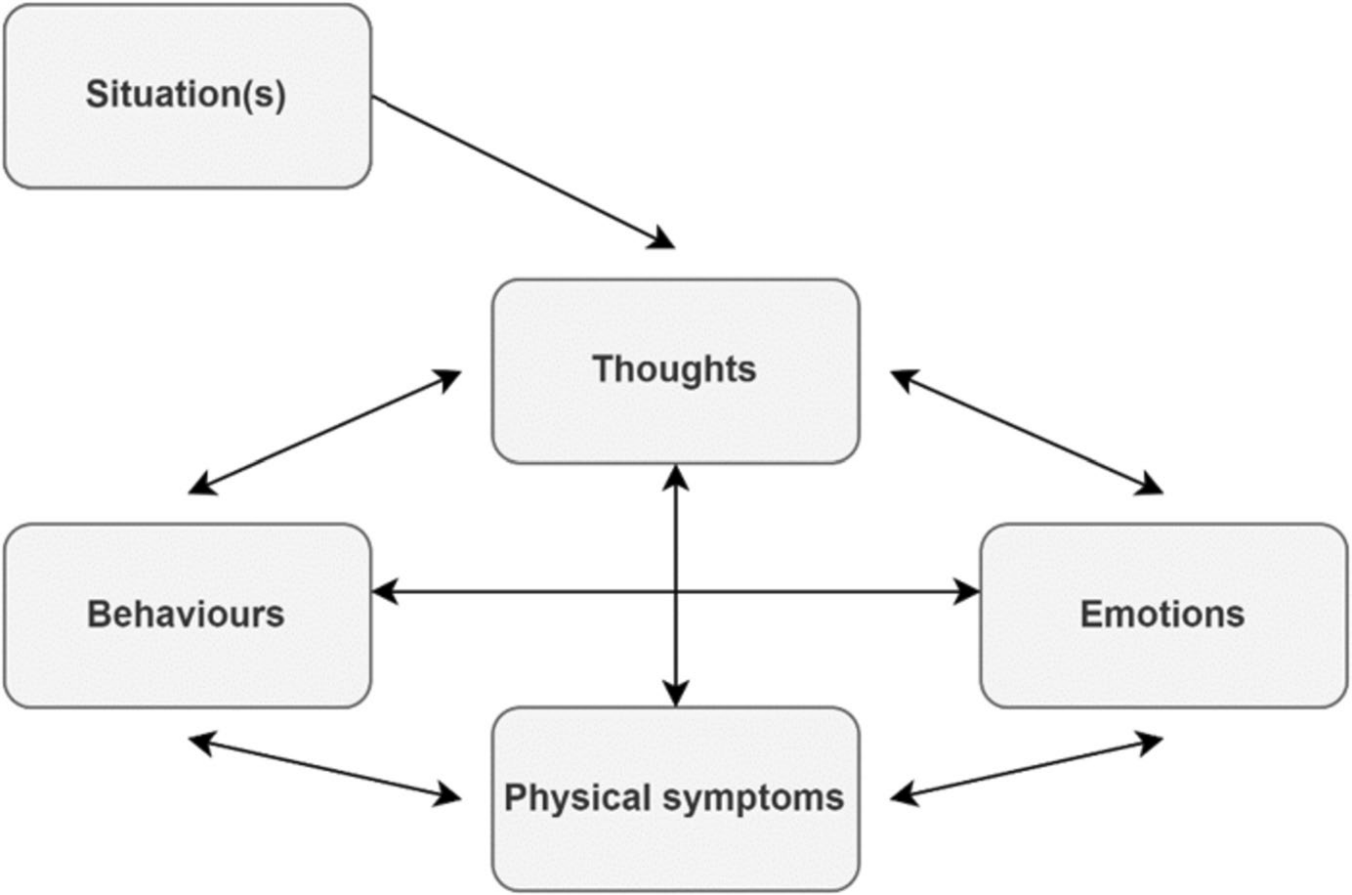
The ‘hot-cross bun’ model of CBT formulation (adapted from Greenberger & Padesky, 1995)

CBT is the most widely used and evidence-supported intervention for common mood (i.e., depression) and anxiety disorders (including social anxiety, generalised anxiety, health anxiety, panic disorder, and obsessive–compulsive disorder) (Beck, 2020). CBT is based upon cognitive-behavioural theories of emotion which suggest that in any given situation, what we think (cognition) and what we do (behaviour) influences how we feel (emotion and somatic sensations). This model is illustrated below in Fig. 1.

There is a considerable evidence base supporting the effectiveness of CBT for the treatment of depression in children and adolescents (see Sigurvinsdóttir et al., 2020). Commensurate evidence does not exist for children with language difficulties. In fact, most researchers explicitly exclude children with verbal IQs below 85 from their clinical trials, with the justification that language difficulties could attenuate treatment effects (James et al., 2020).

### The Language of CBT

Considering the model of CBT (Fig. 1), it is possible to anticipate the challenges associated with this approach for clients like Taylor, with language difficulties. For instance, Taylor would have been required to describe a situation (share a personal story) and verbalise the association between this event and her past and present thoughts, feelings, and/or physical sensations using complex grammar and nuanced vocabulary. She would also be required to engage in hypothetical and future-orientated thinking, articulating possible consequences of her behaviour and evaluate productive outcomes related to thoughts and feelings. Each of these aspects place considerable demand on the use and understanding of language. In this section we consider language demands associated with CBT in greater detail to further highlight the challenges that young people like Taylor may face.

#### A Language Framework

Language is a rule-governed system used to communicate meaning through symbols such as sounds, letters, and words that are expressed by talking, writing, and reading. Though complex, language can be broadly simplified into three key components of content, form, and use (Bloom & Lahey, 1978). Briefly, ‘content’ refers to the meaning of language, where words can be formed correctly and combined correctly in sentences, but if they are not meaningful then messages cannot be conveyed successfully. ‘Form’ refers to the comprehension and production of words that adhere to accepted rules of phonology (the sounds in spoken words), orthography (the letters in written words), grammar (morphology) and sentence structure (syntax). Finally, ‘use’ refers to the way in which language is produced and comprehended in a particular context. Language use is influenced by communication partners (e.g., friend versus colleague), the purpose of communication (e.g., telling a story versus giving a lecture), and includes both higher-level language (such as sarcasm) and non-verbal communicative cues (such as body language and facial expression). This framework is a useful way to identify and describe the discrete language skills utilised in CBT.

##### Language Content in CBT

The language content of CBT is complex. One of the most crucial aspects of CBT is the exploration of Taylor’s thoughts, feelings, and behaviours. In CBT, clients are exposed to, and expected to use, words related to emotions, mental states, biology, mental health, and intervention processes. Emotion vocabulary refers to the range of words and phrases that people use to describe and express their feelings, including more basic emotions such as sadness, anger, and joy, as well as more complex terms to refer to more nuanced internal states such as shame, guilt, or embarrassment. A rich vocabulary of emotion-state words allows an individual to articulate their own and others’ emotions more effectively, enabling better understanding of their own experience(s) and connection with others. Often, vocabulary is used flexibly to express emotions with ambiguous and or figurative language such as, “he is cold” (referring to the presence of hostility rather than the temperature) or “I feel blue” (referring to low mood rather than the colour), respectively. Difficulties with emotion-related vocabulary, ambiguous meanings, and figurative language are commonly associated with DLD (Bahn et al., 2021; Hobson & van den Bedem, 2021). Indeed, even in clients who do not have language difficulties, problems with identifying or describing emotion (a trait known as alexithymia) are associated with increased emotional problems (Çoban & Önder, 2021).

To communicate and understand affective states, individuals need to use mental state vocabulary to refer to the psychological processes to which they are related. Understanding the meaning of terms such as ‘think’, ‘feel’, and ‘believe’, and the differences between them, is essential to communicate emotions, perceptions, and experiences. Psychoeducation involves vocabulary related to the biological causes and effects of mental health conditions such as hormones, chemicals, somatic sensations (e.g., heart rate, sweating, nausea). Additionally, there is vocabulary related specifically to mental health conditions including depression and anxiety, their treatment (e.g., medication), and their similarities and differences in terms of emotional, physical, and/or cognitive symptoms.

CBT sessions also involve a range of instructional processes, in which clients are asked to ‘rate’, ‘quantify’, ‘describe’, and ‘prioritise’; understanding the similarities and differences between these verbs is crucial to respond appropriately. In addition to this ‘universal’ or ‘global’ mental health language, clients are required to utilise specific vocabulary to express the idiosyncratic circumstances related to their individual experiences. In sum, mental health practitioners should consider the complexity of language content used for describing emotions, mental states and instructing clients in session planning and implementation.

##### Language Form in CBT

At a basic level, grammar is critical for representing temporal and causal relationships between thoughts, feelings, actions, and/or events (e.g., why Taylor is feeling depressed). 

Example (client): Yesterday I felt down because I didn’t pass my maths test.

Example (clinician): If you do X, what might/could happen?

Drawing on grammatical knowledge and forms allows us to understand and communicate past, current, future, and hypothetical events, all of which are involved in thinking about and articulating consequences of our own, and others’ actions. Troubles with grammatical knowledge or skills may result in miscommunication of the cause(s) and/or currency of mental health symptoms or experiences, as well as the timeline of salient events or scenarios. The clinician may, therefore, formulate an incomplete — or incorrect — picture of the recency, order, and reality of Taylor’s situation/presenting concern(s). Additionally, grammar facilitates our understanding of, and differentiation between, hypothetical and real concepts or situations. Difficulties with these aspects of grammar may mean the client is unable to relate the hypothetical idea or scenario to themselves to deepen their understanding of their current experience.

##### Language Use in CBT

Using language allows us to reflect on our experiences of ourselves and the world around us and communicate how this impacts our mental health. In the context of CBT, language content and form are used in a range of powerful ways to achieve optimal treatment outcomes. Language is used to build a therapeutic relationship between the client and the clinician. Language allows us to engage in conversation and ‘small talk’ to communicate common interests and build a connection between individuals. The therapeutic alliance is well-established as an important clinical variable, with meta-analytic evidence that stronger therapeutic alliance is associated with positive treatment outcomes (Cameron et al., 2018; Horvath et al., 2011).

During CBT sessions, clients are often required to use language to share autobiographical narratives of past or current events to articulate precipitating situations/scenarios that assist in clinical reasoning. Narratives are the vehicle through which we develop and communicate our understanding of our own and others’ experiences; they are a form of discourse-level language (Spencer & Peterson, 2020). Speakers share a common set of rules for discourse use. Beyond narrative, discourse comes in many forms including persuasion (e.g., persuading a client to adhere to therapy or consider a change), exposition (e.g., explaining the facts about depression to a client), and procedure (e.g., explaining the process of a behavioural experiment, Dipper & Pritchard, 2017; Hill et al., 2021). A client is required to use and engage in a range of discourse forms to express their emotions and experiences, to argue or justify, and empathise with oneself and others. We draw on language skills to identify and repair communication breakdowns, such as may occur when information is misunderstood. Language also supports our ability to request for clarification and advocate for strategies or ways to support communication and participation in conversations.

Language is used during elements of CBT such as modelling or role play where the client is required to adopt the perspective of another person to infer their thoughts or feelings, usually in the context of a hypothetical situation. Socratic Questioning, a core clinical skill in the delivery of CBT, draws heavily on higher-level language skills; requiring the client to carry out and articulate self-reflections, identify and evaluate their thought patterns, and generate and articulate alternative ways of thinking and responding (Padesky, 1993). Often, these questions are open-ended, requiring the client to provide in-depth and critical responses. Language is also fundamental to the delivery of behavioural strategies in CBT. Therapists provide clients with a verbal rationale, engage in planning (e.g., activity scheduling), and post-event processing to consolidate learning (e.g., debrief after exposure).

Language is used to understand, and answer, a range of different types of questions during CBT interactions. There are numerous categories of ‘questions’; most commonly, we are required to ask and answer ‘*wh-*’ questions’ that request information about people (who), things or concepts (what), locations (where), time or timing (when), methods (how), and reasons or causes (why). Not all ‘*wh-*’ questions are equal in terms of their difficulty. Questions that require explanations, reasoning, or analysis are more complex, such as ‘why’, ‘how’ and ‘what if’ questions, are more complex than concrete ‘what’ or ‘when’ questions (Ervin-Tripp, 1970). Questions that ask for causes, reasons, or explanations (why) require deeper understanding of a concept, event, or situation and the ability to articulate the causal or attributive relationships, which typically requires a more complex sentence structure. Questions that inquire about processes or mechanisms (how) draw on understanding of methods, causal relationships, temporal ordering related to concrete or abstract procedures. ‘What if’ questions that explore hypothetical outcomes, events, or reactions (commonly encountered in CBT) are particularly difficult due to their reliance on perspective taking, prediction, and imagination. These question forms are challenging because they require an individual to draw on higher-level language and cognition to reason, synthesise, and infer information. An individual is required to go beyond factual recall (literal information) to engage in deeper and more critical thinking and reflection to provide a meaningful answer. Difficulty with understanding and responding to these more complex question forms has been reported in children with language disorder (Bishop et al., 2006; Deevy & Leonard, 2004).

To summarise, a clear understanding of the various ways in which language is used to comprehend and express emotions, experiences, and thoughts helps shed light on why individuals with language difficulties may struggle to engage in one or a combination of CBT components. By delineating the different uses of language in CBT, it also helps to identify the different tasks that can be supported by strategies to enhance accessibility and support participation.

## Supporting Individuals with Language Difficulties

We argue there are three key pillars to supporting individuals with language difficulties to better access psychotherapeutic interventions, including CBT. These pillars are identifying language difficulties, adapting content and delivery to accommodate language difficulties, and collaborative care between language specialists (SLTs) and mental health practitioners. Like Taylor’s clinician, many therapists may be unaware of a client’s language difficulties. Indeed, a recent study of ‘camouflaging’ in DLD indicates that Taylor may adopt a range of verbal and non-verbal strategies to mask language difficulties from communication partners (Hobson & Lee, 2023). For instance, children with language disorder may nod or say they have understood something when they have not. In addition, some children may use scripts related to specific topics and direct conversation towards topics about which they have adequate familiarity and vocabulary to avoid continued interaction related to complex or unfamiliar topics*.* Taylor may also use non-verbal behaviours like smiling, nodding, and gestures (e.g., pointing) or use ‘distractors’ such as playing on their phone (Hancock et al., 2023). Children with language difficulties may provide minimal responses, or poorly detailed descriptions, or change the conversation towards a preferred topic. In a therapy session, this might seem like a client is disinterested or disengaged, but it can indicate that the client may not have sufficient language to access the discussion or proposed activities. While it is *not* the responsibility of CBT practitioners to formally assess and diagnose language difficulties, practitioners must be aware of some of the indicators of language difficulties, where formal diagnoses are not present. Children may demonstrate slower response time, word-finding difficulties, troubles with temporal ordering of information (e.g., when recalling an event) and/or including key information (e.g., key people or places), as well as difficulty following complex instructions, and understanding or articulating complex or abstract ideas.

Critically, there is little guidance for adapting CBT techniques for children with language disorder (although evidence is emerging; Goh et al., 2023). Therapists may turn to recommended adaptations for CBT in other populations (e.g., people with intellectual disabilities, Surley & Dagnan, 2019); or with aphasia post stroke (e.g., Tjokrowijoto et al., 2024) or may choose to adapt their CBT approach by prioritising behavioural elements of the intervention to reduce language demands (Richards et al., 2016). However, it is unknown whether these approaches are effective for children with language disorder, and the implementation of behavioural strategies is *still* a language-heavy task. For example, clients are still required to articulate and make sense of their experience(s), identify internal thoughts and feelings associated with those experiences, and how these relate to their own and other people’s behaviours or activities. The clinician also describes rationale and procedural information about implementing salient strategies. It is also well established that, to maximise the effectiveness of behavioural strategies, clients are encouraged to engage in cognitive strategies (e.g., reappraisal; Craske et al., 2014). For instance, the principle of expectancy violation suggests clinicians should support clients to engage in processing post-exposure to reconcile the discrepancy between their anticipated/feared outcome, and the observed outcome to maximise their learning (Craske et al., 2014).

Communication Accommodation Theory (CAT; Giles et al., 2023) is a comprehensive theory that, in essence, suggests speakers should modify their communication methods to suit their conversation partner. This theory has been considered in the context of people with other communication difficulties (e.g., Doedens & Meteyard, 2022 & Simmons-Mackie 2018). Simmons-Mackie (2018) discusses the history of CAT and its alignment with Bell’s (1984) concept of audience design, where speakers modify their communication style based on the characteristics of their audience. These accommodations can include changes in speech patterns, language use, pronunciation, timing, loudness, discourse style, and nonverbal communication such as gestures and body language. They can also extend to the modification related to the content of information, the amount of self-disclosure, and the use of humour (Bourhis & Giles, 1977; Giles & Smith, 1979; Gallois & Giles, 2015, as cited in Simmons-Mackie, 2018). Within the context of DLD, one of the most relevant aspects of CAT is ensuring the appropriate level of accommodation for Taylor to participate in conversations. Therapists need to be aware that effective accommodation includes finding the right balance and avoiding inappropriate adjustments. Non-accommodation can occur as either under-accommodation (insufficient adaptation) or over-accommodation (excessive or inappropriate adaptation, Worrall & Hickson, 2003). Striking the right balance in accommodation may significantly enhance communication effectiveness and overall interaction quality (Simmons-Mackie, 2018). In recognition of this, formal supports and interventions have been developed to build the capacity of professionals and other communication partners (e.g., caregivers) to accommodate for the language difficulties associated with DLD (see for example, *Better Conversations with Developmental Language Disorder,* Hughes, 2024) and other neurodevelopmental and acquired conditions (e.g., post-stroke aphasia, Pais & Jagoe, 2023).

To ensure that appropriate accommodations are made, a therapist should first approach with curiosity, asking the child and their supporters (e.g., parents) about their communication preferences, including how information is best presented and how their participation can be effectively supported. When planning and conducting CBT sessions for a child with a language difficulty, it is essential to recognise that the program may take longer (more sessions and/or longer individual sessions), and this should be factored into the therapy schedule where possible. Strategies that lower cognitive load associated with CBT sessions, such as ‘agenda-setting’ (generating an annotated and/or visual plan for each session), can also be beneficial to supporting engagement of children with language difficulties. Another strategy that may be helpful is supporting clients to have a therapy folder or therapy diary, which contains the materials used each week. Clinicians should also consider the complexity of homework prescribed between sessions and provide explicit instructions and/or adapted versions (e.g., emphasise use of visual scales, drawing) where possible. When providing homework for a client to complete between sessions, clinicians should work together to develop an example or template, so that the client has a reference that they can take home. A series of generic strategies that can be used that support language production and comprehension are described in Table 2. We have also provided an example of the implementation of some of these strategies to ‘psychoeducation’ and ‘cognitive restructuring’ as supplementary material. By incorporating these strategies, children like Taylor may more swiftly and effectively engage in mental health support, thereby promoting positive outcomes for these children.

**Table 2:** Strategies to support language comprehension and production during CBT sessions

To support Taylor’s language comprehension:	To support Taylor’s language production:
• Simplify spoken and written information without compromising accuracy. Instructions should be broken down into key components. Readability tools to check the reading level of written materials can be a useful guide • Avoid switching conversation topics quickly. It can be helpful to maintain consistency in conversation topics to allow adequate time to process and respond • Present information in multiple ways using symbols, pictures, and gestures alongside spoken words to help convey key concepts or ideas effectively. For example, using emotion picture cards or age-appropriate YouTube videos to explain the thought-feeling connection • Where possible, use simpler ‘wh-’ question forms to elicit information • Explain key terminology multiple times, with plenty of examples to aid understanding • Speaking slower and allowing ample time to respond to questions may aid comprehension • Teach-Back is an effective method to ensure comprehension. This involves: 1. Using lay terminology to explain key information 2. Asking Taylor to reiterate the information in their own words 3. Re-explaining any misunderstood points 4. Repeating these steps until full understanding is achieved (Talevski et al., 2020)	• Visual supports can be used to scaffold conversation. For example, clinicians may use a visual storyboard (i.e., visual story mountain) to help organise recounts of personal experiences. Picture cards can be helpful to identify when and where an event took place, and how it made the client feel (Zieschank et al., 2021) • While open-ended questions are generally useful for gathering details during sessions (Geldard et al., 2021), they can be difficult for people with language difficulties. Instead, clinicians may ask questions with discrete options alongside open-ended questions where possible. For example, asking ‘Did you feel X or Y?’ instead of ‘How did you feel in this situation?’ • Utilising speech-to-text tools may assist communication more effectively if writing is required. These tools can transcribe spoken language into written text, which can help with clearer organisation and expression of thoughts (Tighe, 2022) • Write down a list of the client’s key points. Read these back to identify information that is missing, misunderstood, or requires additional detail (i.e., a supported Teach-Back strategy)

These suggestions have been informed by a broad research literature (including studies of child language disorder and other clinical populations that experience language difficulties [e.g., post-stroke aphasia]). Members of the authorship team have received funding to co-design guidelines to enhance language-based accessibility of mental health interventions specifically for children with language difficulties

**Fig. 2:**
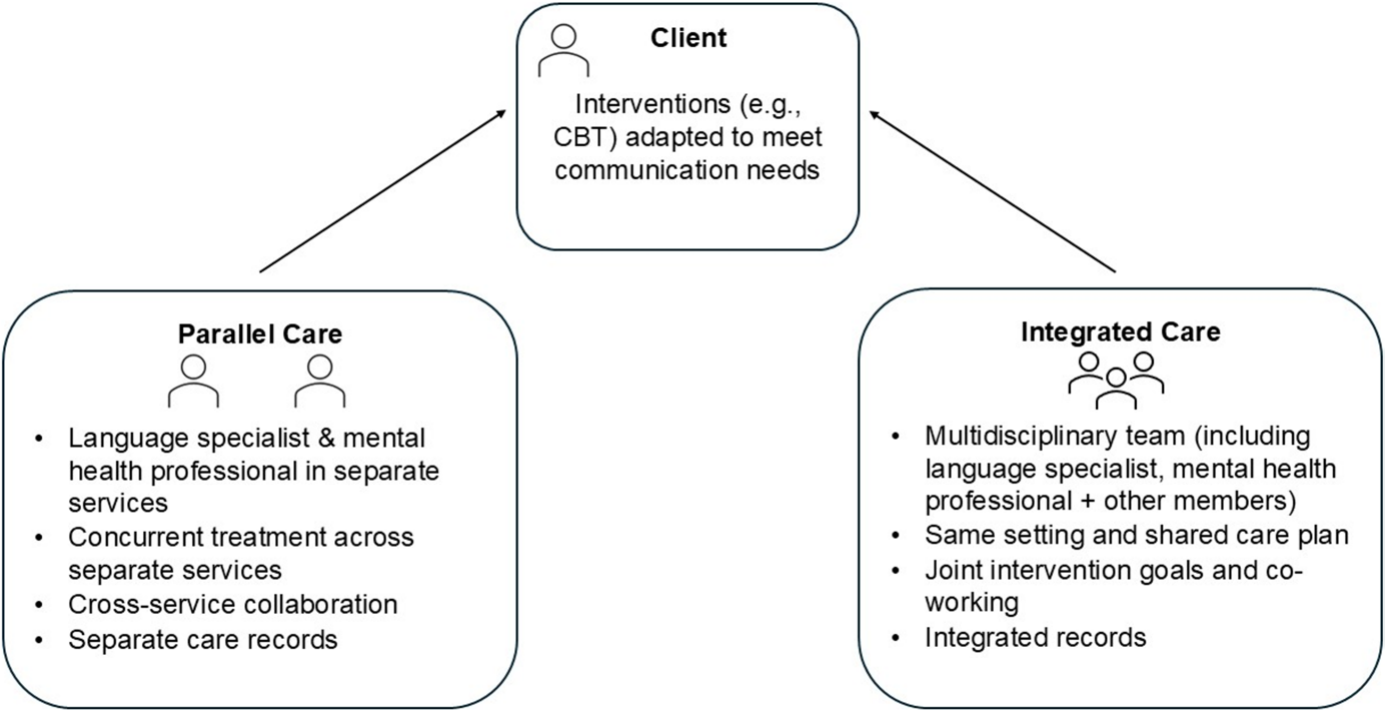
A proposed model of collaborative care between mental health professionals and language specialists (i.e., SLTs) informed by Boon et al. (2004) and Reist et al. (2022)

Implementing ad hoc strategies to support communication is not a long-term solution to the inaccessibility of mental health services to children with low language skills. SLTs and mental health professionals must work together to optimise access to, and engagement in, mental health support for these children. SLTs have a role in providing language intervention, raising awareness of language difficulties, and can build the capacity of mental health professionals to support communication in practice (Hancock et al., 2023; Hill et al., 2024). Mental health professionals can up-skill SLTs in the identification of mental health difficulties and the intersection of language and mental health problems. Multidisciplinary education in pre-service training - driven by collaboration between experts in language and mental health - would be a critical enabler of timely and accessible mental health support for children with language difficulties. Figure 2 provides a visual representation of how a collaborative care process could operate when supporting individuals with co-occurring mental health and language difficulties. Practitioners may view collaborative care as a process in which professionals from different disciplines come together to develop a unified treatment plan, drawing on their collective expertise to address the varied needs of the clients such as Taylor (Boon et al., 2004). We present this figure to begin the conversation on how such services can be implemented, but we recognise that translating these practices from research into routine clinical care is challenging and requires further research to strengthen its evidence base (Reist et al., 2022).

## Conclusion

Through Taylor’s experience, we have illustrated the potential inaccessibility of CBT for children with language difficulties by describing the barriers to engagement and specifically unpacking the language content, form, and use involved in ‘talking therapy’. Taylor’s scenario is a stark reality for many children with language disorder who experience inequitable mental health services. While we have chosen to focus this article on DLD as an exemplar, many other populations live with co-occurring language and mental health difficulties for whom adaptations to CBT would be beneficial. Recognising that participation in CBT can be a complex task for individuals with language difficulties is a crucial step toward developing meaningful accommodations. The recommendations provided here are pragmatic and can be applied broadly in scenarios where language barriers, irrespective of underlying origin, necessitate extra support. Indeed, there is some evidence for the efficacy and acceptability of modified CBT in adults with post-stroke aphasia (Tjokrowijoto et al., 2024). We suggest that mental health professionals should be cognisant of the language demands associated with their practice and advocate for effective and equitable support for people living with language difficulties. We conclude with recommendations to identify language difficulties, adapt psychotherapy for individuals with language difficulties, and highlight the importance of collaborative care between mental health professionals and language specialists. Formal evaluation of the effectiveness of these strategies, the development of adapted psychotherapies, and models of collaborative care for people living with language difficulties remain an urgent priority.

## Supplementary Information

Below is the link to the electronic supplementary material. 

ESM1 (DOCX 19.7 KB)

## Data Availability

Data availability does not apply to this article as no new data were created or analysed in this study.
