# Supplementary material for: “Why Would Someone like Me with DLD Want to Sit in a Room and Talk? How Would that Make Me Feel Better?!” Developmental Language Disorder and the Language Demands of Cognitive Behaviour Therapy
Source: Int J Cogn Behav Ther. 2025 May 27;18(3):405–24. doi: 10.1007/s41811-025-00254-3 (PMC12496285; doi:10.1007/s41811-025-00254-3)
Supplement: Supplementary file 1 — (DOCX 19.7 KB) [file 41811_2025_254_MOESM1_ESM.docx]

| **Supplementary material –** Example script and potential adaptations across two components of CBT (psychoeducation and cognitive restructuring). Numbers correspond to script elements and corresponding descriptors. | | | | |
| --- | --- | --- | --- | --- |
| **CBT component** | **Example script (excerpts of clinician dialogue only)** | **Description of language-based complexity** | **Simplified script (excerpts of clinician dialogue only)** | **Description of language-based adaptation** |
| Psychoeducation | “It might be helpful for us to start by talking about what depression is. What do you know about depression? (1)”  “Depression is a common and serious mental health condition that affects how you feel, think (2), and handle (3) daily activities. It’s more than just feeling down or going through a rough patch (4).”  “Depression can lead to various symptoms, which can be grouped into emotional, cognitive, physical, and behavioural. Let's break these down. Emotional symptoms include feeling ‘blue’, or empty feelings (4), persistent (5) sadness, feelings of hopelessness, feelings of guilt, or helplessness (6).  Depression can manifest in physical symptoms like changes in sleep patterns, appetite changes, fatigue or lack of energy, and various aches and pains (7).” | (1) Open-ended question  (2) No explicit definition of key concepts (e.g., ‘feel’ and ‘think’)  (3) Atypical / colloquial verb (“handle”)  (4) Non-literal language (e.g., idiomatic expression ‘rough patch’; ‘empty’ feeling’; feeling ‘blue’)  Complex / low frequency vocabulary:  (5) adjectives (e.g., ‘persistent’)  (6) emotions terminology (e.g., ‘hopelessness’, ‘guilt’)  (7) symptomology without definition (e.g., ‘symptoms’, ‘sleep patterns’, fatigue’, ‘aches and pains’ | “Have you heard of depression before?” [*yes/no*] (1)”  “Depression happens to a lot of people (2). Depression can change your feelings (3).”  “Feelings are what we feel inside (4). We can feel happy. We can feel sad (5)” [*optional support with visual aid, e.g., emotion cards*].”  “Depression can change your thoughts. Our thoughts help us understand things (4). We use our thoughts to answer questions and make decisions (5).”  “Depression can make you feel different ways. It can change your feelings, thoughts, and your body (2).”  “Depression can make you feel very sad. You can feel like you have done something wrong (2). You can feel angry (5). Sometimes you might not know how to feel better. (2)”  “Depression can change the way your body feels (2,3). You might sleep more. You might not be hungry. You might feel tired (5).” | (1) Modify question type based on client’s response (*if unable to answer open-ended move towards guided or close-ended question*)  (2) Define key terms with simplified vocabulary  (3) Simplified sentence structure (*subject* + *verb* + *object*)  (4) Defining key concepts separately and after first mention.  (5) Providing concrete examples.  ***Additional adaptations:***  Speaking at slower rate  Use of visual support  Clinician may confirm Taylor’s comprehension via Teach Back  Where feasible, arrange longer and/or additional sessions to facilitate a slower pace (acknowledging organisational and funding constraints)  Cover one concept per session (e.g., depression only) |
| Cognitive restructuring | “The goal is to identify and challenge your negative thoughts (1) and replace them with more realistic ones. Sound good? Ok Taylor, let’s start by examining the thought, ‘*Lily didn’t text me. She must hate me*.’ Tell me more about what happened (2) and why you think this way (3)”  “On a scale of 0 to 100, how strongly did you believe this thought [that Lily must hate you] at the time? (4)”  “What evidence (5) do you have that supports this thought?”  “There are often multiple ways to interpret the same event. Let’s reframe your original thought into a more realistic one (6). What would be a different way to think about Lily’s message? (7)”  “Do you think it is helpful to think this way about Lily? (1), (8)”  ***““***Over the next week, it would be really helpful for you to write down some thoughts that we could look at next week together. If you notice some of those uncomfortable or difficult emotions, make a note in the thought diary (just the first few columns - what was happening, what you felt, and what you were thinking). Then we can explore it in more detail next time.” | (1) Assumes understanding of “thought” and “realistic” versus “unrealistic” thoughts.  (2) Clinician requests personal narrative  (3) “Why” requires response that addresses the underlying cause. Necessitates complex sentence (e.g., use of complex conjunction ‘because’ to indicate causal or temporal relationship between event(s) and thoughts).  (4) Use of rating scale to quantify abstract concept (quantifying belief). Requires Taylor to reflect on past feeling (“at the time”).  (5) Assumes understanding of “evidence” as supporting fact(s) related to the thought.  (6) Requires critical judgment of the value / meaning of an idea or event or concept and whether it aligns with – or rebuts – thought.  (7) Requires hypothetical thinking, perspective taking (i.e., multiple interpretations of one event).  (8) Complex and abstract concept of a thought being helpful or unhelpful  (9) Thought diary relies heavily on written language for completion. May not be explicit overview of instructions or examples. | Now, we will talk about your thoughts. Thoughts help us understand things. We use our thoughts to answer questions and make decisions (1). We want to change our thoughts to make us feel better.  You said that Lily didn’t text you back. You think this means that Lily does not like you (2). Let’s think about if this thought is true. Something is true when it is based on fact (2).  “Now let’s think about *how* you know that it is true. Remember, true means it is based on fact (1). Let’s think about what clues we have that Lily feels this way (3). A clue is some information that helps us work out if something is true or not (1). Clues help us solve a problem or answer a question (4). Our clues could be something Lily has said or something Lily has done. We can write down our clues here (5) [*Clinician may use sticky notes or plain paper* *to note down ideas*].”  “You said that Lily has not sent you a text back. What are some reasons why somebody may not reply to a text? I will go first. Somebody could forget to reply…(6, 7)”  “A helpful thought is something that makes us think differently about problems. A helpful thought about Lily might be that she was busy, or she had something else going on. A thought that is not helpful would be that she has not texted you back because she does not like you. Can you think of another helpful thought? (7, 8)”  “Before I see you next, I want you to write down some thoughts in this diary. We will talk about these thoughts next week. Remember a thought help us understand something (9). We use our thoughts to answer questions and make decisions. You can fill this out when you feel a negative emotion – like sad or angry. Today, you told me you think Lily does not like you. Lily didn’t text you back. This is a good example of a negative thought (10). When you feel a negative emotion like sad or angry, write or draw about this in the diary. We can look through this next week.  “Can you tell me what the plan is for the next week? (11)” | (1) Clear definition of key concept(s). Clinician may need to repeat definition multiple times within and between sessions.  (2) Use simple sentences to express action and consequence (causal relationship between events).  (3) Introduce a new task with a statement to direct attention and introduce key concept(s).  (4) Use simplified or more high frequency vocabulary (e.g., ‘clue’ for ‘evidence’).  (5) Use multimodal ways of expressing information.  (6) Provide clear example(s).  (7) Clinician steps out Socratic question  (8) Complex question (helpful/unhelpful thoughts) explained with explicit definition and example(s).  (9) Reminder of key concepts / definitions  (10) Concrete example to model homework  (11) Clinician utilises Teach Back strategy  (12) Clinician works with the client to ‘bullet point’ the plan for the subsequent session. Client can take a photo of the plan as a reminder and to facilitate discussion with supports (e.g., caregivers). |
| *This table includes two CBT techniques. The purpose of this table is to provide illustrated examples of the potential language-based complexity of psychoeducation and cognitive restructuring and adaptations that can be made to support language production and comprehension.* | | | | |
